# Associations between environmental quality and infant mortality in the United States, 2000–2005

**DOI:** 10.1186/s13690-018-0306-0

**Published:** 2018-10-15

**Authors:** Achal P. Patel, Jyotsna S. Jagai, Lynne C. Messer, Christine L. Gray, Kristen M. Rappazzo, Stephanie A. Deflorio-Barker, Danelle T. Lobdell

**Affiliations:** 1 Oak Ridge Institute for Science and Education at the U.S. Environmental Protection Agency, National Health and Environmental Effects Research Laboratory, Chapel Hill, NC USA; 2 School of Public Health, Division of Environmental and Occupational Health Sciences, University of Illinois, Chicago, IL USA; 3 OHSU-PSU School of Public Health, Portland State University, Portland, OR USA; 4 Gillings School of Global Public Health, Department of Epidemiology, University of North Carolina, Chapel Hill, NC USA; 5 U.S. Environmental Protection Agency, National Health and Environmental Effects Research Laboratory, MD 58A, Research Triangle Park, NC 27711 USA

**Keywords:** Multiple environmental exposures, Environmental quality, Air quality, Water quality, Land quality, Built environment, Sociodemographic environment, Infant mortality

## Abstract

**Background:**

The United States (U.S.) suffers from high infant mortality (IM) rates and there are significant racial/ethnic differences in these rates. Prior studies on the environment and infant mortality are generally limited to singular exposures. We utilize the Environmental Quality Index (EQI), a measure of cumulative environmental exposure (across air, water, land, sociodemographic, and land domains) for U.S. counties from 2000 to 2005, to investigate associations between ambient environment and IM across maternal race/ethnicity.

**Methods:**

We linked 2000–2005 infant data from the U.S. Centers for Disease Control and Prevention to the EQI (*n* = 22,702,529; 144,741 deaths). We utilized multi-level regression to estimate associations between quartiles of county-level EQI and IM. We also considered associations between quartiles of county level domain specific indices with IM. We controlled for rural-urban status (RUCC1: urban, metropolitan; RUCC2: urban, non-metropolitan; RUCC3: less urbanized; RUCC4: thinly populated), maternal age, maternal education, marital status, infant sex, and stratified on race/ethnicity. Additionally, we estimated associations for linear combinations of environmental quality and rural-urban status.

**Results:**

We found a mix of positive, negative, and null associations and our findings varied across domain and race/ethnicity. Poorer overall environmental quality was associated with decreased odds among Non-Hispanic whites (OR and 95% CI: EQIQ4 (ref. EQIQ1): 0.84[0.80,0.89]). For Non-Hispanic blacks and Hispanics, some increased odds were observed. Poorer air quality was monotonically associated with increased odds among Non-Hispanic whites (airQ4 (ref. airQ1): 1.05[0.99,1.11]) and blacks (airQ4 (ref. airQ1): 1.09 [0.9,1.31]). Rural status was associated with increased IM odds among Hispanics (RUCC4-Q4:1.36[1.04,1.78]; RUCC1-Q4: 1.04[0.92,1.16], ref. for both RUCC1-Q1).

**Conclusions:**

This study is the first to report on associations between ambient environmental quality and IM across the United States. It corroborates prior research suggesting an association between air pollution and IM and identifies residence in thinly populated (rural) areas as a potential risk factor towards IM amongst Hispanics. Some of the counterintuitive findings highlight the need for additional research into potentially differential drivers of environmental quality across the rural-urban continuum, especially with regards to the sociodemographic environment.

**Electronic supplementary material:**

The online version of this article (10.1186/s13690-018-0306-0) contains supplementary material, which is available to authorized users.

## Background

Infant mortality is the death of a baby before their first birthday and is frequently used as a global indicator of health and well-being [1]. Despite considerable healthcare spending and continual advancements in medicine and public health, the United States (U.S.) suffers from one of the highest infant mortality rates among the world’s developed nations [2, 3]. In 2013, the overall infant mortality rate was 5.96 per 1000 live births. For infants of Non-Hispanic white mothers, the rate was 5.06 per 1000 births; that rate was roughly similar amongst Hispanic mothers at 5.0 per 1000 births but nearly double for Non-Hispanic black mothers at 11.11 per 1000 births [2, 4]. A better understanding of factors contributing to infant mortality and the observed racial/ethnic disparity remains an important public health target in the United States.

Adverse environmental exposures during fetal development and infancy are risk factors for poor health outcomes. This is because fetal development is mediated through maternal factors and maternal exposure to environmental agents has been shown to induce genetic alterations and developmental deficits in the fetus [5]. Developing infants are particularly susceptible to the state of their environment on account of their immature defense systems and their low body mass [6, 7]. Even at the preconception stage, environmental exposures can adversely impact the reproductive systems in both sexes, which may contribute downstream to compromised infant health [8, 9]. While prior work has considered how environmental factors influence birth outcomes, the role of cumulative environmental exposures as determinants to infant health is an area calling for additional research.

Commonly, studies exploring the relationship between environmental exposure and infant mortality focus on single exposures such as arsenic or particulate matter [10, 11]. In instances when multiple exposures are considered, they tend to fall under the same general environmental construct (e.g., multiple air pollutants). Single-exposure environmental studies have great utility in identifying modifiable exposures; however, by their very nature, they fall short of providing a comprehensive assessment of potential environmental risk. For instance, the risk posed by residence in an area where there is exposure to particulate matter from industrial operations may be further exacerbated by simultaneous exposure to pesticides and area level poverty. Such a scenario presents negative environmental factors working in tandem, but there is also growing recognition that there are positive, health-promoting environmental factors (e.g. parks, grocery stores) that occur simultaneously with negative factors to create an environmental exposure profile that drives health outcomes [12, 13].

The Environmental Quality Index (EQI) provides a cumulative environmental exposure for counties in the United States [14, 15]. The EQI was generated through a two-step principal components analysis, in which more than 200 variables falling under five key environmental domains (air, water, land, built, and sociodemographic) were empirically reduced to generate domain-specific indices, which were further reduced to generate an overall environmental quality index [14, 15].The EQI can therefore be utilized to examine the larger environmental context in association with infant mortality. In assessing the relationship between environmental quality and infant mortality, an important consideration is rural-urban residence. Rural residence is a potential risk factor for infant mortality in the United States [16, 17]. A recent report indicated significant differences in cause-specific (ex. congenital malformations, sudden infant death syndrome) infant mortality rates across rural and urban strata in the U.S. for the years 2011–2013 [16]. Additionally, environments differ across urban-rural status and different environmental exposure profiles may drive environmental quality in urban versus rural areas [18]. Thus, exploration into effects corresponding to combinations of levels of environmental quality and rural-urban status is warranted.

To our knowledge, no published studies have examined the relationship between cumulative environmental exposures and infant mortality, accounting for rural-urban status and potential modification by maternal race/ethnicity. We address this gap in the literature through a cross-sectional analysis using the county EQI and Centers for Disease Control and Prevention (CDC)‘s linked birth infant death data for the years 2000–2005. Our analyses are stratified by maternal race/ethnicity to evaluate potential effect modification and allow for qualitative comparisons of the role of environmental quality in the observed racial/ethnic disparities in infant mortality rates in the United States.

## Methods

### Infant mortality outcome data

Infant mortality was defined as death before completion of first year of life [1]. We obtained linked birth and infant death data from the U.S. Centers for Disease Control and Prevention for the years 2000–2005, corresponding to the time frame covered by the EQI. Prior to any exclusions, there were 24,490,885 infant records, of which 162,643 experienced deaths. We identified five racial/ethnic groups, namely Non-Hispanic white, Non-Hispanic black, Hispanic, Asian, and American Indian/Alaskan Native (AI/AN) to examine modification by race/ethnicity. Because there were too few Asian and AI/AN infants in most counties for stable estimates, these were excluded (*n* = 1,673,044 records, 10,073 deaths). Additionally, we excluded infants born to women whose Federal Information Processing Standard (FIPS) codes did not match up to one of the 3141 counties included within the EQI (102,661 records excluded, 534 deaths), as well as infants that experienced accidental or violent death (7295 records excluded, Additional file 1: Table S1). Lastly, we excluded 5356 records that were doubly included on account of data format shift from birth-cohort to period-linked from 2002 onwards. The final study population comprised 22,702,529 infants, 144,741 of whom died before completing their first year of life.

### Environmental quality index (EQI) and domain specific exposure data

The EQI served as our primary exposure and it represents cumulative environmental quality at the county level for the years 2000–2005 in the contiguous U.S. Both the conceptual framework and the methodology underlying the EQI have been previously published [14, 15]. As a brief overview of EQI construction, five environmental domains (air, water, land, built, and sociodemographic) were identified and data on representative variables for each domain were collected. Principal component analysis was used to reduce the variables representing these five environmental domains into domain specific indices; these five indices were then included in a second principal component analysis to generate an overall environmental quality index [19]. Each PCA resulted in loadings (measure of correlation between variable and principal component) for input variables, which were then integrated into a linear combination model in conjunction with standardized input variable values to generate the composite indices [19]. Broadly, higher EQI values represent worse environmental quality. All of the EQI data were obtained from U.S. Environmental Protection Agency (EPA) and linked to the outcome data using county of residence at death (0.53%), county of death occurrence (~ 0%), county of residence at birth (99.39%), and county of birth occurrence information (0.09%), in that order of availability. Both the overall EQI and the five domain-specific indices were modeled as quartiles, with the first quartile indicating best quality and the fourth indicating worst.

### Covariates

Rural-urban status was included as a co-exposure in our models, defined by rural-urban continuum codes (RUCC) from the U.S. Department of Agriculture [20]. Consistent with prior literature, we collapsed the nine groups into four: metropolitan urbanized (RUCC1), non-metropolitan urbanized (RUCC2), less urbanized (RUCC3), and thinly populated (RUCC4) [21–23]. Potential confounders included maternal age (<=19 years, 20–29 years, 30–39 years, and 40+ years), maternal education (less than high school completed, high school completed, or greater than high school completed), marital status (married/unmarried), and maternal race/ethnicity (Non-Hispanic white, Non-Hispanic black, and Hispanic). We also included infant sex as a strong predictor of our outcome variable.

### Statistical analysis

Our objective was to assess the relationship between county-level environmental exposures, as captured by the overall EQI and domain-specific indices, and individual level infant mortality across maternal race/ethnicity. For each maternal race/ethnicity category, we constructed two separate, fully-adjusted random intercept, fixed slope multilevel models, the first with overall EQI as the primary exposure and the second including all domain specific indices as primary exposures. Post modeling, linear combinations of coefficients for the overall EQI/domain specific indices and rural-urban status (RUCC) variables were computed. We conducted two supplementary analyses, one in which we considered the relationship between EQI/domain-specific indices and infant mortality in the absence of RUCC and another in which we considered the relationship between RUCC and infant mortality in the absence of EQI/domain specific indices (all other co-variates were included in both models). We performed all analyses using Stata 14 and report all findings in the form of odds ratios and corresponding 95% confidence intervals.

## Results

### Study population characteristics

Our study population comprised 22,702,529 infants born in the United States between the years 2000–2005, of whom 144,741 died (6.4 infant deaths per 1000 live births). There were 13,869,745 infants born to Non-Hispanic white mothers, 3,484,425 born to Non-Hispanic black mothers, and 5,348,359 born to Hispanic mothers. Of the infants born to Non-Hispanic white mothers, 73,003 died (5.2 infant deaths per 1000 live births); 44,046 (12.6 infant deaths per 1000 live births) and 27,692 (5.1 infant deaths per 1000 live births) infant deaths were observed among Non-Hispanic black and Hispanic mothers, respectively. Differences in proportions of maternal rural-urban status, level of maternal education, marital status, and maternal age were observed for the study population across the maternal race/ethnicity categories and across the infant mortality outcome variable (Table 1).

**Table 1:** Study population characteristics by maternal race/ethnicity and infant mortality (IM) status, United States (2000–2005)

Characteristics	All (*N* = 22,702,529)		Non-Hispanic White (*N* = 13,869,745)		Non-Hispanic Black (*N* = 3,484,425)		Hispanic (*N* = 5,348,359)	
Infant deaths	144,741		73,003		44,046		27,692	
% mothers experiencing IM	0.64		0.53		1.3		0.52	
	No IM	IM	No IM	IM	No IM	IM	No IM	IM
Married (%)	65	51	77	66	31	27	55	50
Maternal education (%)								
Missing (n = 2,394,404)								
> High School	42	32	52	40	33	28	19	16
High School	28	31	26	31	35	35	27	26
< High School	20	24	10	17	22	23	43	44
Maternal age (%)								
< =19 years	11	16	8	13	18	19	15	18
20–29 years	52	52	40	50	56	54	57	52
30–39 years	34	29	40	34	24	25	26	26
40+ years	2	3	3	4	2	2	2	3
Male (%)	51	56	51	57	51	56	51	56
Rural-Urban status (%)^a^								
RUCC 1	84	84	80	77	90	89	93	92
RUCC 2	7	7	8	9	5	5	4	4
RUCC 3	8	8	10	11	5	6	3	4
RUCC 4	1	2	2	2	< 1	< 1	< 1	< 1

^a^RUCC 1: metropolitan urbanized areas, RUCC 2: non-metropolitan urbanized areas, RUCC 3: less urbanized, RUCC 4: thinly populated areas

### Association between EQI and infant mortality

**Fig. 1:**
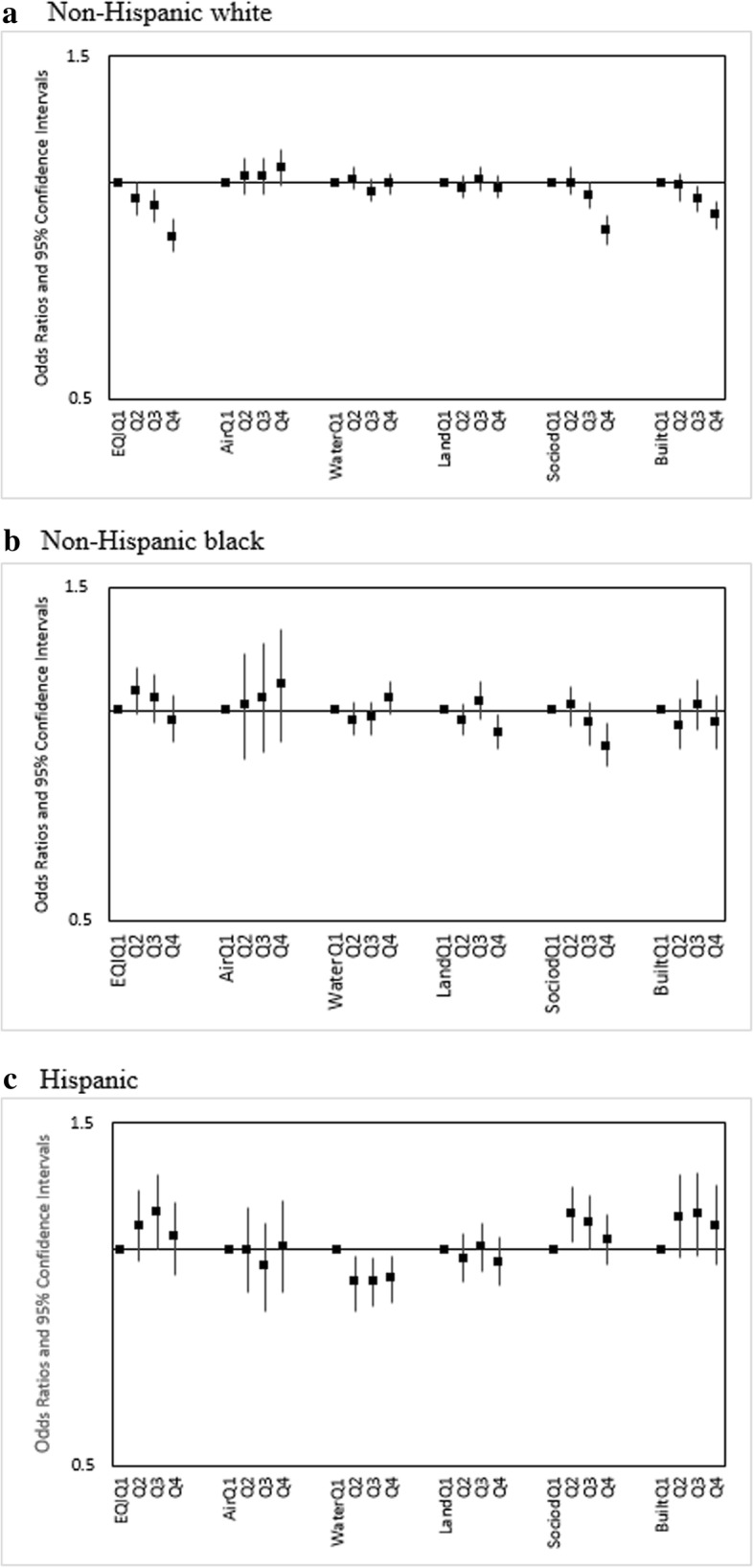
Adjusted infant-mortality ORs (95% CI) for overall EQI and domain-specific indices across **a** Non-Hispanic White, **b** Non-Hispanic Black, and **c** Hispanic mothers, United States (2000–2005). Both the overall EQI and the five domain-specific indices were modeled as quartiles, with the first quartile indicating best quality and the fourth indicating worst

We found different trends in association between increasing EQI (i.e. poorer environmental quality) and infant mortality across our maternal race/ethnicity categories. We observed decreased odds of mortality among infants of Non-Hispanic white mothers (OR and 95% CI: Q2: 0.95 [0.91, 1], Q3: 0.93 [0.88, 0.98], Q4: 0.84 [0.8,0.89]) (Fig. 1a). For infants of Non-Hispanic black and Hispanic mothers, we observed some increased odds, although the trends in association differed as follows: (OR and 95% CI: Q2: 1.07 [0.99, 1.15], Q3: 1.04 [0.96,1.13], Q4: 0.97[0.9, 1.05]) for infants of Non-Hispanic black mothers (Fig. 1b) and (OR and 95% CI: Q2: 1.08 [0.96, 1.21], Q3: 1.13 [1.00, 1.27], Q4: 1.04 [0.92, 1.16]) for infants of Hispanic mothers (Fig. 1c).

**Fig. 2:**
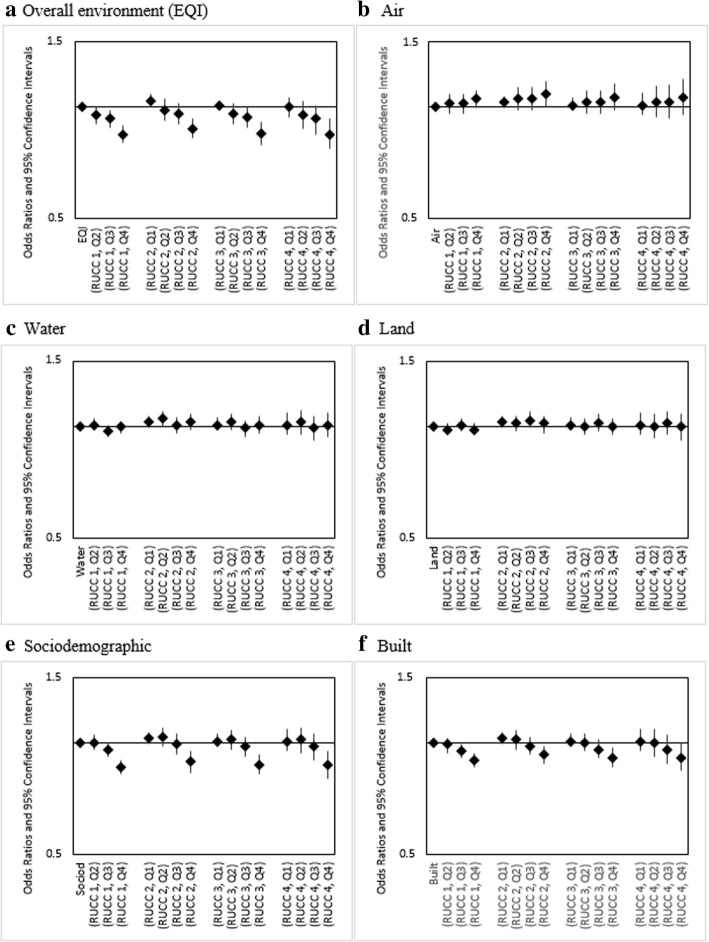
Adjusted ORs (95% CI) for linear combinations of overall EQI/domain-specific indices and rural-urban status for infants of Non-Hispanic White mothers, United States (2000–2005). RUCC 1: metropolitan urbanized areas, RUCC 2: non-metropolitan urbanized areas, RUCC 3: less urbanized, RCC 4: thinly populated areas. Both the overall EQI (**a**) and the five domain-specific indices (**b-f**) were modeled as quartiles, with the first quartile indicating best quality and the fourth indicating worst

**Fig. 3:**
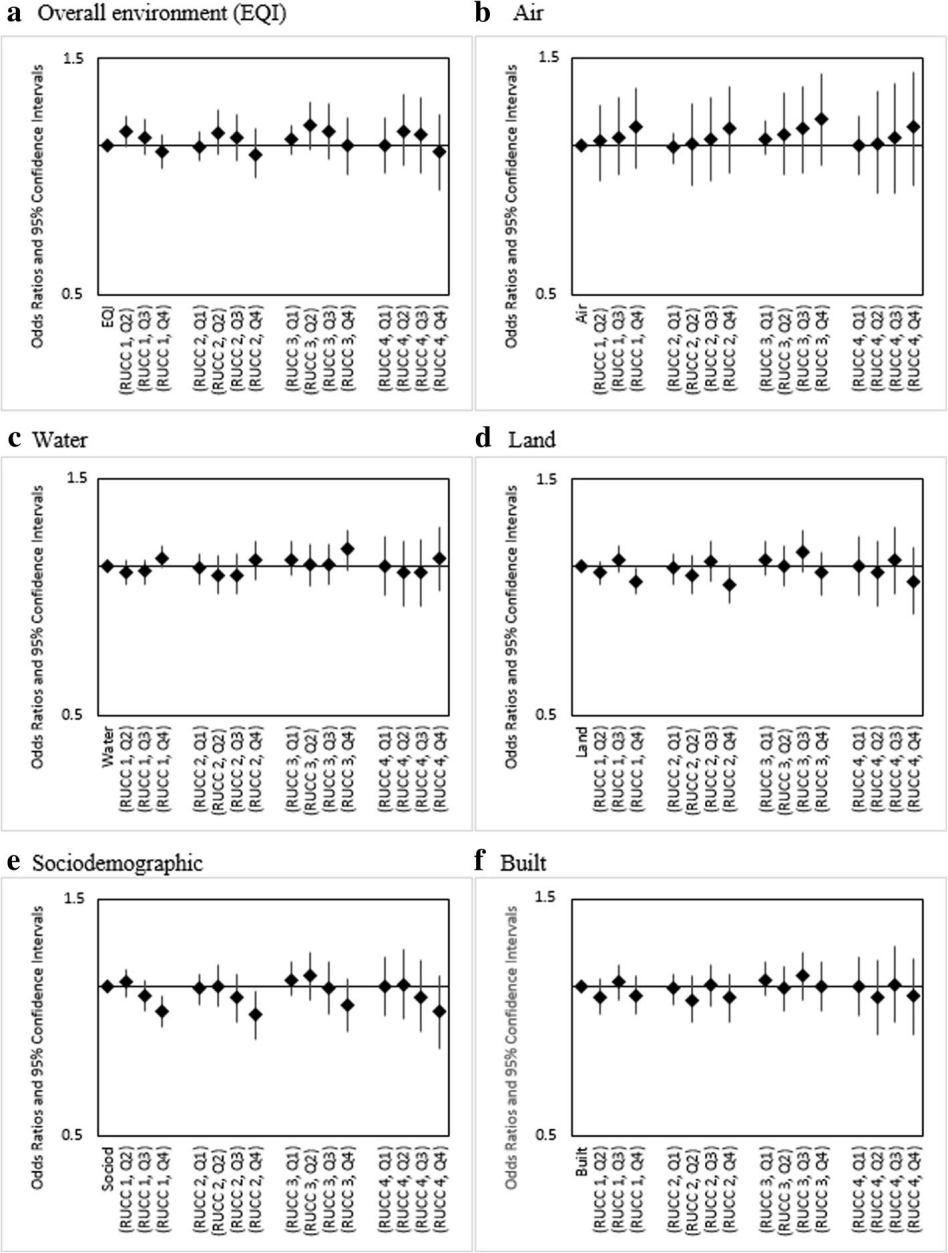
Adjusted ORs (95% CI) for linear combinations of overall EQI/domain-specific indices and rural-urban status for infants of Non-Hispanic Black mothers, United States (2000–2005). RUCC 1: metropolitan urbanized areas, RUCC 2: non-metropolitan urbanized areas, RUCC 3: less urbanized, RUCC 4: thinly populated areas. Both the overall EQI (**a**) and the five domain-specific indices (**b-f**) were modeled as quartiles, with the first quartile indicating best quality and the fourth indicating worst

**Fig. 4:**
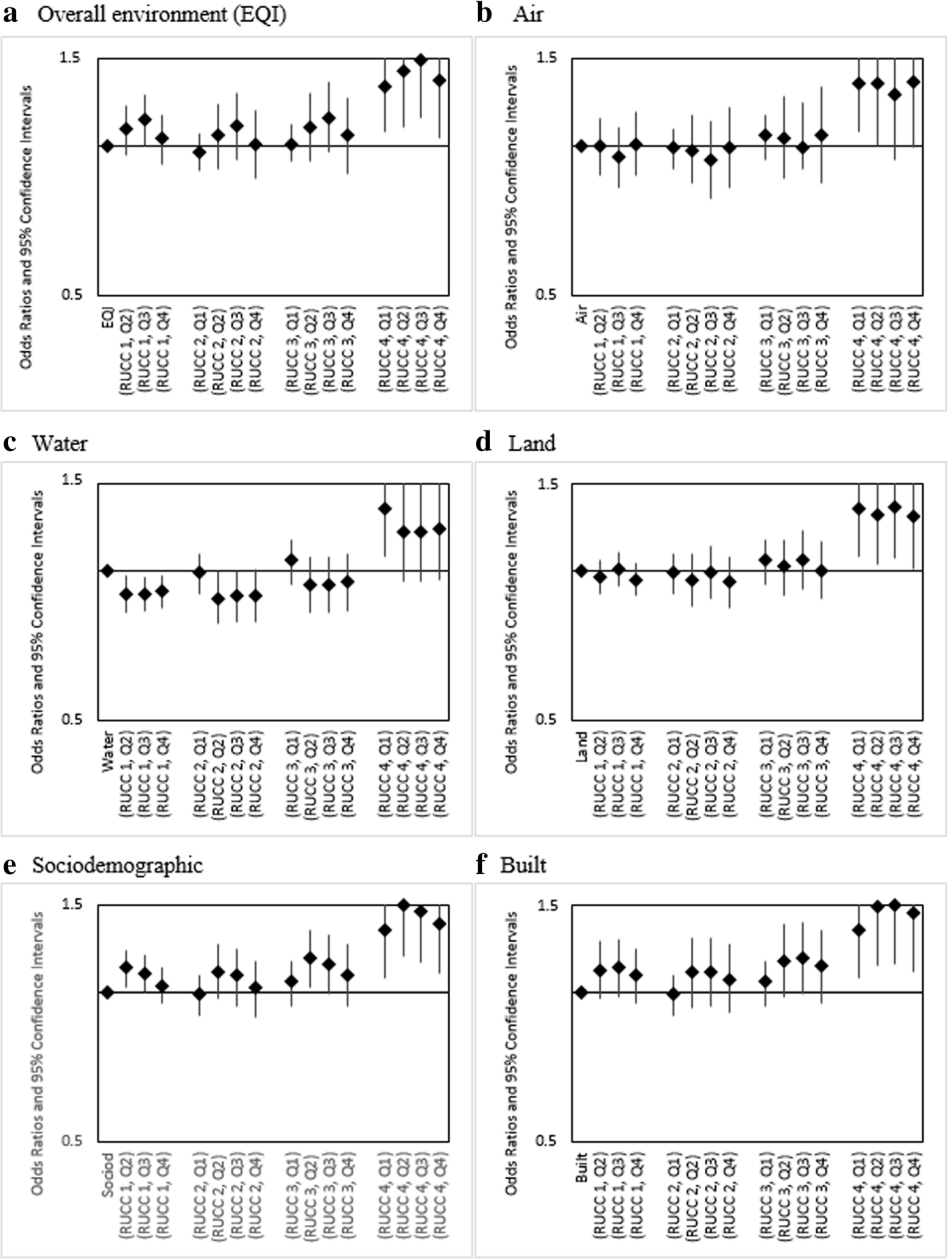
Adjusted ORs (95% CI) for linear combinations of overall EQI/domain-specific indices and rural-urban status for infants of Hispanic mothers, United States (2000–2005). RUCC 1: metropolitan urbanized areas, RUCC 2: non-metropolitan urbanized areas, RUCC 3: less urbanized, RUCC 4: thinly populated areas. Both the overall EQI (**a**) and the five domain-specific indices (**b-f**) were modeled as quartiles, with the first quartile indicating best quality and the fourth indicating worst

For linear combinations of EQI and RUCC, for which RUCC1-Q1 (urbanized metropolitan, best environmental quality) served as the referent group, we observed variations across our maternal race/ethnicity categories. For infants of Non-Hispanic white (Fig. 2a-f) and Non-Hispanic black mothers (Fig. 3a-f), rural-urban status was observed to have little impact on infant mortality risk. As an example, for Non-Hispanic white, the combinations RUCC1-Q4, RUCC2-Q4, RUCC3-Q4, and RUCC4-Q4 all showed decreased odds within the narrow range of 0.84–0.87. Among Hispanic mothers, increasing rurality was associated with increased infant mortality odds for all levels of environmental quality. For instance, RUCC4-Q4 showed increased odds (1.36 [1.04, 1.78]) compared to RUCC1-Q4 (1.04 [0.92, 1.16]) (Fig. 4a-f).

### Association between domain-specific indices and infant mortality

We observed monotonic, increasing infant mortality odds for increasing air index (poorer air quality) among Non-Hispanic white (airQ4 (ref. airQ1): 1.05[0.99,1.11]) and Non-Hispanic black mothers (airQ4 (ref. airQ1): 1.09 [0.9,1.31]) (Fig. 1a, b). No association was observed among infants of Hispanic mothers (Fig. 1c). Combinations of air index and RUCC showed similar trends to those observed for combinations of EQI and RUCC; rural status was associated with increased infant mortality odds among Hispanic mothers while having slight to no impact among Non-Hispanic white and Non-Hispanic black mothers (Figs. 2a-f, 3a-f, 4a-f).

Across water and land domains, we observed null to slightly decreased odds for increasing water and land indices and infant mortality among Non-Hispanic white and Non-Hispanic black mothers (Fig. 1a, b). Among infants of Hispanic mothers, consistent decreased odds were observed (Panel 1c). For combinations of these respective domain indices and RUCC, the associations were either slightly positive, null, or slightly negative, with no apparent trends across domain quality or rural-urban status. The exceptions were RUCC-Q1, RUCC4-Q2, RUCC4-Q3, and RUCC4-Q4 among Hispanics, where consistently increased odds were seen (Fig. 4a-f).

For sociodemographic and built domains, decreasing odds were seen with increasing domain indices among Non-Hispanic white mothers (Fig. 1a). Among Non-Hispanic black mothers, we saw mostly decreased and some slightly increased odds (Fig. 1b). With Hispanics, we saw some increased odds, but the relationship between the domain indices and infant mortality was not monotonic as observed for the air domain (Fig. 1c). Combinations of the domain indices and RUCC showed similar trends to those observed for combinations of EQI and RUCC (Figs. 2a-f, 3a-f, 4a-f).

## Discussion

The primary aim of this study was to explore the relationship between environmental quality and infant mortality across maternal race/ethnicity. To more fully describe the role of rural-urban status in this relationship, we estimated linear combinations of environmental quality/rural-urban status and infant mortality. We found that independent and combined effects of environmental quality and rural-urban status on infant mortality varied across domain and by race/ethnicity.

The finding that decreasing environmental quality is monotonically associated with decreasing infant mortality odds among Non-Hispanic white mothers is counter-intuitive, as are trends in association among Non-Hispanic black and Hispanic mothers. A possible explanation lies in that the effect of environmental insults on health is also dependent on the health status / accumulated stressors of the affected population. Non-Hispanic white women are generally exposed to fewer of these stressors than Non-Hispanic black and Hispanic women, so the effect of poor environments may be insufficient to overcome their relatively privileged health status [24].

Another potential explanation lies in the structure of the EQI. Across our maternal race/ethnicity categories, decreasing sociodemographic quality was generally associated with decreasing infant mortality odds, often monotonically. This is in contrast to much of existing literature, where associations between poor sociodemographic factors and adverse pregnancy outcomes have been documented [25, 26]. It is plausible that the trends in association in the sociodemographic domain, and to a lesser extent, the built domain, are driving the relationships observed for overall environmental quality. Unlike air, water, and land domains, which are comprised primarily of contaminants and toxicants, many of the variables that comprise the sociodemographic and built domains may be more heterogeneous in their impact across the urban-rural continuum. For instance, the sociodemographic variables median household income, median household value, percent persons under poverty level, and percent unemployed are potentially differential predictors of adverse health outcomes such as infant mortality in the urban versus rural setting due to differences in the cost and style of living. This is reflected in the loading patterns from the principal components analysis for these variables.

In construction of the EQI, underlying variables were assigned a positive value if known or thought to be associated with adverse health outcomes; with the percent unemployed variable, for instance, the RUCC1, RUCC2, RUCC3, and RUCC4 specific loadings are + 0.3718, − 0.4053, − 0.3429, and − 0.3322 respectively [15]. The overall loading (all RUCC considered simultaneously) for this variable is − 0.3250. This is to be expected given that we are leveraging information across all U.S. counties and roughly 70% of those counties (RUCC2, RUCC3, RUCC4) have negative loadings as noted above [15]. The negative overall loading value means that higher values of percent unemployed contribute to moving the sociodemographic index in the negative (better quality) direction (index values were obtained through sum of product of variable loadings and corresponding county level standardized values), enabling some of the aforementioned counterintuitive associations. From a broader perspective, the marked urban-rural discrepancies in sociodemographic variable loading patterns suggest that available measures of sociodemographic quality may be more relevant in urban contexts than in rural contexts. Greater understanding of what defines relative socioeconomic deprivation within rural areas continuum and inclusion of potential novel drivers would add to the strength of the sociodemographic index and by extension the overall EQI in the future.

We observed associations in the expected direction in the air domain: decreasing air quality was associated with increasing infant mortality odds among Non-Hispanic white and Non-Hispanic black mothers, and the observed relationship was monotonic. Air domain variables such as PM_10_, carbon disulfide, and vinyl chloride are comparatively more consistent markers for air quality and more consistent predictors of adverse health outcomes across the urban-rural continuum, as indicated by the positive loadings for these and other air domain variables across RUCC categories and the overall United States [15]. Our findings are consistent with previous studies that investigated air pollutants and infant health. Ritz et al. evaluated the impact of CO, NO_2_, PM_10_, and O_3_ (criteria air pollutants) exposure on infant health in the South Coast Air Basin of California, a region with one of the worst air quality in the United States [27]. The authors considered exposures 2-weeks, 1-month, 2-months, and 6-months prior to death, reporting positive associations for CO_2_, NO_2_, and PM_10_. Another study by Woodruff et al. covered 86 Metropolitan Service Areas (MSAs) within the United States and focused specifically on particular matter pollution, reporting a 4% increased odds (OR and 95% CI: 1.04[1.02–1.07] in all-cause infant mortality for a 10 microgram/m^3^ increase in particulate matter concentration [28]. To our knowledge, no previous studies have examined composite indices of air quality in association with infant mortality. This study adds to the body of literature examining air pollution and infant mortality as we were able to capture exposure to numerous hazardous air pollutants (in addition to commonly examined criteria pollutants) and describe a monotonic association across the United States as opposed to only urban, metropolitan areas. Moreover, in evaluating the relationship between air quality and infant mortality, we considered all five environmental domains simultaneously, which accounts for simultaneous exposure to factors across other facets of the environment. This modeling structure may be a possible explanation for why we observed no association between worsening air quality and infant mortality among Hispanic mothers. It may be that in controlling for sociodemographic and built environment quality, both of which were positively associated with infant mortality, the impact of air quality among Hispanic women was attenuated. It is important to note that our estimates among Hispanics were comparatively less precise because of the relative rarity of infant mortality amongst Hispanics compared to Non-Hispanic blacks) and the heavily urban-metropolitan (RUCC1) biased distribution of the Hispanic population. Our findings should be viewed within this context.

In post-estimation analysis of linear combinations of environmental quality and rural-urban status, we observed that rural residence was associated with elevated infant mortality odds among Hispanic mothers. For instance, infant mortality odds were 32% higher for RUCC4-EQIQ1 compared to RUCC1-EQIQ1, and similar relationships were observed for individual domains. Probst et al. reported that in 1999–2000, 44.9% of working-age rural Hispanics were uninsured, compared to 31.9% and 17.8% for rural Non-Hispanic black and white, respectively [29]. Moreover, rural Hispanics were more likely to be uninsured across both the working-age and child age groups relative to their urban counterparts. In their study, Probst et al. also found that roughly 80% of all rural counties where Hispanics were the majority population (more than half the population of the county) were whole or partial health professional shortage areas. While health care infrastructure is captured to an extent within the built environment domain of the EQI, rural-urban differences in insurance status and other potential access barriers may be a possible explanation for the observed association between rural residence and infant mortality among Hispanic mothers, independent of environmental quality. Moreover, as mentioned previously, interpretations of the impact of rural residence among Hispanics should be made within the context of rural-urban distribution of this population and precision of estimates.

This study has several limitations. Our exposure data is at the county level, and this may not be the best geographic unit for data aggregation for variables in particular environmental domains especially if there is potential for high heterogeneity in individual exposure [15]. For instance, exposure to land pollutants such as radon can vary substantially across individual home sites. Similarly, individual exposure to water contaminants and recreational water may vary substantially across individuals in a county. This may be a potential explanation for the largely null associations observed in the land and water domains. Future investigation with data at finer spatial scales such as the census tract level will inform this possibility. Moreover, our analysis is cross-sectional in nature, with the EQI representing average environmental quality over the six-year time period of 2000–2005. There is potential for variation in some of our considered environmental factors over this time frame; however, we expect the overall quality, especially at the county level, to be relatively stable. Previous studies reporting on environmental exposures and infant mortality identify mainly short-term associations (e.g., air pollutants and sudden infant death syndrome); therefore, we believe our choice of a cross-sectional analysis to be appropriate [27, 28].

This study has several strengths. To our knowledge, it is the first to investigate an association between cumulative environmental exposure and infant mortality in the United States. It demonstrates methodological feasibility and potential for the use of environmental indices that capture broad environmental context and multiple facets (domains) of the environment when examining its relation to health outcomes on large geographic scales. The results, while limited by some of the domain-specific uncertainties and counterintuitive findings, suggest potential racial/ethnic differences in the impact of aspects of the environment on infant mortality. Moreover, the indication that available sociodemographic markers may capture urban and rural contexts differently may be informative toward future iterations of measures such as the EQI and research at the intersections of environment, rural-urban status, and health outcomes.

## Conclusions

This study was the first to investigate the association between an index of cumulative environmental quality and infant mortality across the United States. We stratified our analysis by maternal race/ethnicity and post-estimation considered linear combinations of environmental quality and rural-urban status. We observed positive, negative, and null associations across our analyses. Among the domains, poor air quality was positively associated with infant mortality among Non-Hispanic white and Non-Hispanic black mothers. The sociodemographic and built domains were negatively associated for Non-Hispanic white, mostly negative to null for Non-Hispanic black, but were positively associated among Hispanics. The effect of residence in thinly populated (rural) areas was found to be most acute among Hispanics, independent of environmental quality. This research is not without limitations, with constraints due to domain specific uncertainties and the county-level unit of exposure assessment. We also identify the need for additional research into drivers of environmental quality across the rural-urban continuum, with particular regard to the sociodemographic environment.

## Additional file

Additional file 1: **Table S1.** Description of data: ICD-10 codes utilized to exclude accidental/violent death. (DOCX 12 kb)

## Abbreviations

AI/AN: American Indian/ Alaskan native
CDC: Centers for disease control and prevention
CO_2_: Carbon dioxide
EPA: Environmental protection agency
EQI: Environmental quality index
FIPS: Federal information processing standard
IM: Infant mortality
MSA: Metropolitan service area
NO_2_: Nitrogen dioxide
PM_10_: Particulate Matter (10 μm or less in diameter)
RUCC: Rural-urban continuum code
U.S.: United States
