# Supplementary material for: Associations between environmental quality and infant mortality in the United States, 2000–2005
Source: Arch Public Health. 2018 Oct 15;76:60. doi: 10.1186/s13690-018-0306-0 (PMC6191999; doi:10.1186/s13690-018-0306-0)
Supplement: Supplementary file 1 — Table S1. Description of data: ICD-10 codes utilized to exclude accidental/violent death. (DOCX 12 kb) [file 13690_2018_306_MOESM1_ESM.docx]

Table S1. ICD-10 codes utilized to exclude accidental/violent death.

| **Code** | **Description** |
| --- | --- |
| V01-V99 | Transport accidents |
| W00-W19 | Falls |
| W32-W34 | Accidental discharge of firearms |
| W65-W74 | Accidental downing and submersion |
| W75 | Accidental suffocation and strangulation in bed |
| W76-W77, W81-W84 | Other accidental suffocation and strangulation |
| W78-W80 | Accidental inhalation and ingestion of food or other objects causing obstruction of respiratory tract |
| X00-X09 | Accidents caused by exposure to smoke, fire, and flames. |
| X40-X49 | Accidental poisoning and exposure to noxious substances |
| W20-W31, W35-W64, W85-W99,  X10-39, X50-X59 | Other and unspecified accidents |
| X91 | Assault (homicide) by hanging, strangulation and suffocation |
| U01.4, X93-X95 | Assault (homicide) by discharge of firearms |
| Y06-Y07 | Neglect, abandonment and other maltreatment syndromes |
| U01.0-U01.3, U01.5-U01.9, X85-X90, X92, X96-X99, Y00-Y05, Y08-Y09 | Assault (homicide) by other and unspecified means |
| Y40-Y84 | Complications of medical and surgical care |
